# Prediction of Lycii Cortex Quality Marker Based on Network Pharmacology and Chemometrics Methods

**DOI:** 10.1155/2024/1790697

**Published:** 2024-10-22

**Authors:** Xinrui Wang, Guotong Li, Haoqiang Ding, Xiqin Du, Lanying Zhang, Jingze Zhang, Dailin Liu

**Affiliations:** ^1^State Key Laboratory of Component-Based Chinese Medicine, Tianjin University of Traditional Chinese Medicine, Tianjin 301617, China; ^2^TCM Formula R&D Department, Tianjin Modern Innovation Chinese Medicine Technology Co., Ltd., Tianjin 300380, China

**Keywords:** chemometrics, Lycii Cortex, Q-markers, UPLC–MS/MS, UPLC–Q-TOF-MS

## Abstract

Based on the effectiveness, measurability, and traceability of the quality marker (Q-marker) theory of traditional Chinese medicine, the Q-marker of Lycii Cortex (LC) was preliminarily predicted and analyzed. A UPLC–Q-TOF-MS qualitative analysis method for LC samples was established. A total of 44 LC chemical components, 16 plasma prototype components, 25 urine prototype components, and 27 fecal prototype components were identified. At the same time, the “component–target–disease” network diagram was constructed by network pharmacology to predict the potential active components of LC. A UPLC–MS/MS quantitative analysis method was established to determine the contents of 11 components such as kukoamine A in 35 batches of LC from seven producing areas. Principal component analysis, orthogonal partial least squares discriminant analysis, and other mathematical analysis methods were used to screen the differential components. Based on the comprehensive consideration of the Q-marker traceability, transitivity, specificity, effectiveness, and measurability, kukoamine A and kukoamine B were preliminarily predicted as LC potential Q-markers, and the high-quality producing area was determined to be Chengcheng County, Weinan City, Shaanxi Province. The prediction analysis of the LC Q-marker provides a reference for the comprehensive control of the quality of LC medicinal materials and also lays a foundation for the research and exploration of the substance basis and mechanism of action of LC.

## 1. Introduction

Lycii Cortex (LC) was initially documented in the Western Han Dynasty in the “52 Diseases Formula.” Based on traditional Chinese medicine (TCM) principles, the LC is classified under the lung meridian. It is renowned for its therapeutic effects, including clearing heat, detoxifying, nourishing yin, moistening dryness, reducing inflammation, and stopping bleeding. Yam deficiency is commonly used to alleviate lung heat symptoms. Modern pharmacological research has shown that LC exhibits various activities, including hypotensive, hypoglycemic, antibacterial, antiviral, antipyretic, and antiparoxysmal effects. The active ingredients of LC mainly consist of alkaloids and organic acids, which have drawn the attention of pharmacologists. Recent research has focused on alkaloid compounds, which have shown promise in the clinical treatment of conditions such as pneumonia, lung cancer, diabetes, and Alzheimer's disease [1–3].

Due to the influence of the geographical environment, harvesting, and storage, the composition of compounds in LC and their pharmacological effects are different, resulting in uneven LC quality. According to the 2020 edition of the Chinese Pharmacopoeia, the source of Cortex Lycii is clearly the dry root bark of Lycium chinense Mill. or Lycium barbarum L. However, there is no established quality standard for LC, which raises questions about the selection of high-quality drugs and the identification of premium production regions. Therefore, the establishment of methods for determining the content is crucial for researching high-quality medicinal materials and identifying regions with optimal production, which will ensure effective quality control [4]. In 2016, the academician Changxiao Liu in China proposed the concept of a quality marker (Q-marker) based on five core principles: traceability and transitivity, specificity, measurability, effectiveness, and prescription compatibility. By screening chemical substances closely related to the safety and effectiveness of TCM, this process provides a reference for the quality control of Chinese herbal medicine.

In recent years, scholars have begun to study LC content determination methods. Zhang et al. established a high-performance liquid chromatography (HPLC) coupled with electrospray ionization (ESI) tandem mass spectrometry method for 24 components in LC [5] and developed a method for structural characterization of phenolic amides from LC by ultrahigh-performance liquid chromatography coupled with linear ion trap orbitrap tandem mass spectrometry [6]. Zhao et al. established a characteristic RSLC fingerprint with 14 common peaks [7]. Li et al. used kukoamine A and kukoamine B as Q-markers and established a quality control method for LC using HPLC [8]. Although many scholars have conducted extensive research, the specificity and effectiveness of the measured indicators are still unclear. Therefore, it is extremely necessary to establish comprehensive and effective quality control methods. Network pharmacology [9] is a research method that utilizes the correlation between composition and efficacy as a Q-marker. This technique is used to explore the mechanism of multicomponent and multitarget TCM treatment. It has been widely used in the study of Q-markers for various Chinese herbal medicines, Chinese herbal pieces, and Chinese herbal compounds. The pattern recognition method in chemometrics is the key method to screen the Q-markers of TCM. Pattern recognition technology mainly includes principal component analysis (PCA), cluster analysis, partial least squares (PLS) method, etc., which can reduce the dimension of high-dimensional data, improve the identification of fingerprints, and accurately classify the functions and characteristics of medicinal materials. In this paper, network pharmacology combined with chemometrics methods were used to analyze and process the data. According to the traceability, effectiveness, and measurability of Q-markers, the Q-markers of LC were screened out, which laid a scientific foundation for the LC quality control and evaluation model.

**Figure 1:**
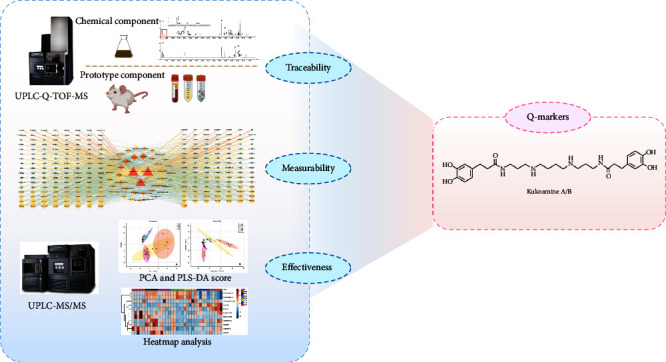
Flowchart of the experiment.

Based on the effectiveness, measurability, and traceability of the Q-marker, the chemical components in LC and the prototype components in the plasma, urine, and feces of rats after oral administration of LC were identified by UPLC–Q-TOF-MS technology. The “component–target–disease” network was constructed by network pharmacology to predict potential Q-markers in LC. UPLC–MS/MS was used to determine the concentrations of 11 potential markers, and the concentrations of 35 batches of LC from seven producing areas were determined. Finally, multivariate statistical analysis was used to analyze the high-quality producing areas of LC. The results of this study could provide a reference for the quality control and evaluation of LC and lay the foundation for further research. The workflow of this study is presented in Figure 1.

## 2. Materials and Methods

### 2.1. Chemicals and Reagents

Eleven pure compounds were used as reference standards (purity ≥ 98%). Among these compounds, guanosine (111977-201501), chlorogenic acid (110753-201817), caffeic acid (110885-201701), ferulic acid (110773-201614), kaempferol (110861-202013), luteolin (111520-202006), apigenin (111901-202004), and emodin (110756-201913) were purchased from the China Consummate Testing International Academy of Inspection and Monitoring (Beijing, China). Kukoamine A (ST82040105, 7314), kukoamine B (ST79760105, 7315), and scopolin (ST05010120, 7317) were purchased from Shanghai Shidande Standard Technical Service Co., Ltd. (Shanghai, China).

### 2.2. Animals

Eighteen male Wistar rats weighing 180–220 g supplied by SPF (Beijing) Biotechnology Co., Ltd. (License Number: SCXK (Beijing, China) 2019-0010 and Animal Certificate Number: 1112511911000098) were kept under specific pathogen-free (SPF)-grade conditions (22°C ± 2°C, a natural light–dark cycle, a relative humidity of 50 ± 10%) and provided water and food ad libitum. The animals were acclimatized to the facilities for 1 week and then fasted with access to water for 12 h prior to each experiment. The animal experiments were performed in accordance with the Guidelines for the Care and Use of Laboratory Animals, and the study protocol was reviewed by the Institute of Radiation Medicine, Chinese Academy of Medical Sciences (Reference Number: IRM-DWLL-2022135).

One hundred grams of LC was weighed, 10 times the amount of water was added to extract for 1 h, the sample was filtered, 8 times the amount of water was added for 40 min, the sample was filtered, the filtrate was combined twice, the sample was concentrated to 100 mL, and the sample was stored in a refrigerator at −4°C for later use. Eighteen Wistar rats were randomly divided into a blank group, a plasma group, and a urine/feces group, with six rats in each group. Except for the blank group, which was given physiological saline by gavage, the other groups were given LC drug solution by gavage at a dose of 2.43 g·kg^−1^.

### 2.3. Sample Preparation

#### 2.3.1. Preparation of LC Solution

The medicinal materials of the LC were removed, crushed, and passed through a No. 3 sieve. The powder (0.1 g) was placed in a 25-mL conical bottle with a stopper. Then, 25 mL of 70% methanol was added, and the mass was accurately measured. Ultrasonic treatment (power: 250 W, frequency: 40 kHz) was conducted for 30 min. After cooling, the sample was reweighed, and 70% methanol was added to minimize mass loss. The mixture was shaken well and centrifuged at 12, 470 × g for 10 min. The supernatant was collected and filtered through a 0.22-*μ*m microporous membrane to obtain the test solution.

#### 2.3.2. Preparation of Plasma, Urine, and Feces Solutions for UPLC–Q/TOF-MS

In the plasma group, after fasting but with free access to water for 12 h, the rats were orally administered drugs. Blood samples were collected from the eye vein plexus at 15, 30, and 60 min and 2, 4, 8, 12, and 24 h after administration. The samples were placed in 1% sodium heparin anticoagulant tubes and centrifuged at 4°C and 3000 × g for 10 min, after which the supernatant was stored at −80°C. The plasma samples from each time point were combined, and 200 *μ*L of the mixed plasma was placed in a centrifuge tube, followed by the addition of 1 mL of methanol. After vortexing for 3 min, the mixture was centrifuged at 4°C and 12, 470 × g for 10 min. The supernatant was transferred to another clean centrifuge tube, blown dry with nitrogen gas, 100 *μ*L of methanol was added for resuspension, the tube was vortexed for 3 min and centrifuged, and the supernatant was collected for analysis.

Urine/feces group: Rats were housed in metabolic cages with free access to water but not food. The test substance was orally administered, and urine and feces were collected at 0–4, 4–8, 8–12, 12–24, and 24–48 h and stored at −80°C for later use. Urine and feces samples from each time point were pooled separately, and urine samples were prepared in the same manner as plasma samples. One gram of dried feces was extracted by ultrasonic extraction with 1 mL of methanol for 60 min and centrifuged at 12, 470 × g for 10 min, after which the supernatant was collected for sample analysis. Blank samples were processed in the same way to obtain plasma, urine, and feces.

### 2.4. UPLC–Q/TOF-MS Conditions

LC was separated on a Waters CORTECS T3 column (2.1 × 150 mm, 1.6 *μ*m). The mobile phase consisted of 0.1% (*v/v*) formic acid in water (Solvent A) and acetonitrile (Solvent B). The gradient elution program was set as follows: 0–3 min, 0% ⟶ 2% B; 3–10 min, 2% B; 10–12 min, 2% ⟶ 5% B; 12–18 min, 5% B; 18–40 min, 5% ⟶ 8% B; 40–55 min, 8% ⟶ 28% B; 55–65 min, 28% ⟶ 100% B; 65–70 min, 100% B; 70–72 min, 100% ⟶ 0% B; and 72–80 min, 0% B. Mass spectrometry was performed using an ESI source.

Positive and negative ion switching modes in the range of m/z 100–1500 were used for MS analysis. The ion source parameters were capillary voltages of 3.00 kV (ESI^+^) and 2.50 kV (ESI^−^), a sampling cone voltage of 50 V, a source temperature of 120°C, and a cone gas flow rate of 50 L·h^−1^. The desolvation gas was heated to 800°C and delivered at a gas flow rate of 550 L·h^−1^. The parameters were set to be the same in both positive and negative ion modes. The reference solution was 200 pg·*μ*L^−1^ leucine enkephalin, and real-time correction was performed at a flow rate of 10 *μ*L·min^−1^.

### 2.5. Component–Target–Disease Network Construction

Through the Traditional Chinese Medicine Systems Pharmacology Database and Analysis Platform (TCMSP) (https://tcmsp-e.com/tcmsp.php), the compounds and targets of the TCM LC were identified. The active ingredients and targets were screened for oral bioavailability (OB) ≥ 30% and drug likeness (DL) ≥ 0.18. Combined with a literature search, the active ingredients were preliminarily determined. The SwissTargetPrediction (https://www.swisstargetprediction.ch/) and HERB (https://herb.ac.cn/Search/) databases were used to assist in identifying supplementary targets. All targets were retrieved from the UniProt database (https://www.uniprot.org/), and nonhuman targets were excluded using the filter “Homosapiens.” After summarizing and removing duplicates, the compound components and the total number of targets were obtained.

The GeneCards database was used to obtain genes related to the efficacy of LC treatment. The keywords used were “relieving fever,” “cough with lung heat,” and “hypoglycemic rash.” Scores greater than the median of the relevant scores were combined to eliminate repetitive items, and the targets related to the efficacy of LC were obtained as disease targets.

The LC component targets and the LC efficacy targets were imported into the Venn diagram online platform (https://bioinformatics.psb.ugent.be/webtools/Venn/) for the intersection. The “LC” and “Effect” were used to indicate the efficacy of LC and LC, respectively. The intersection was used to identify the common targets, and a Venn diagram was created.

Finally, the component–target–disease pathway network was established and visualized using Cytoscape 3.9.1 software.

### 2.6. UPLC–MS/MS Conditions

An Acquity H-Class UPLC and a Xevo TQ-S Micro triple quadrupole mass spectrometer equipped with an ESI interface were used in this study (Waters Corp., Milford, MA, USA). Twenty-four analytes were separated using an Acquity UPLC BEH Shield C18 column (2.1 × 100 mm, 1.7 *μ*m), and the column temperature was set at 40°C. The mobile phase consisted of 0.1% formic acid and acetonitrile at a flow rate of 0.3 mL/min. The gradient elution conditions were as follows: 0–8 min, acetonitrile changed from 5% to 95%; 8–9 min, acetonitrile decreased to 5%; and 9–13 min, acetonitrile was maintained at 5%.

**Figure 2:**
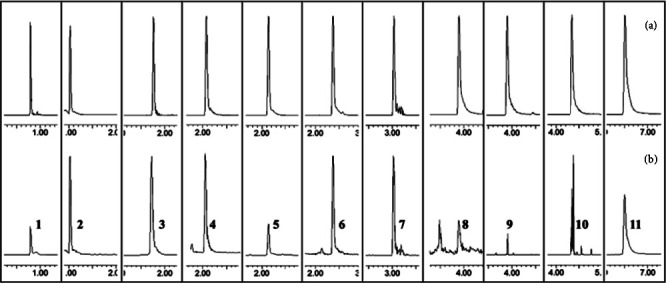
MRM mass spectra of 11 index components. (a) Standard solution and (b) LC solution. (1) Kukoamine A, (2) guanosine, (3) kukoamine B, (4) chlorogenic acid, (5) scopoline, (6) caffeic acid, (7) ferulic acid, (8) kaempferol, (9) luteolin, (10) apigenin, and (11) emodin.

The desolvation and cone gases were set at 800 L/h and 150 L/h, respectively. Helium was selected as the collision gas. The capillary voltage was set to 2.5 kV. The source and desolvation temperatures were both set to 400°C. Multiple reaction monitoring and positive/negative ESI modes were used for quantitative analysis [10] (Table 1, Figure 2).

### 2.7. Method Validation

According to the 2020 edition of the Chinese Pharmacopoeia, the method of multi-index content determination, including accuracy, precision, linearity, range, repeatability, stability, and sample recovery, was verified.

### 2.8. Content Determination

LC was purchased from Tianjin Modern Innovation Traditional Chinese Medicine Technology Co., Ltd. The sources of these batches were identified by Professor Guo Baolin from the Institute of Medicinal Plants, Chinese Academy of Medical Sciences. A total of 35 batches of LC were divided into seven categories according to the different producing areas and suppliers. Then, the test solution was prepared, analyzed, and determined according to the conditions of liquid chromatography‒mass spectrometry, the peak area of each component was recorded, and the contents of guanosine, chlorogenic acid, caffeic acid, ferulic acid, kaempferol, luteolin, apigenin, emodin, kukoamine A, kukoamine B, and scopolin in different batches of ground bark samples were calculated. Information on the origin of the medicinal materials is given in Table 2.

## 3. Results

### 3.1. Identification of the Prototype Components of LC

**Figure 3:**
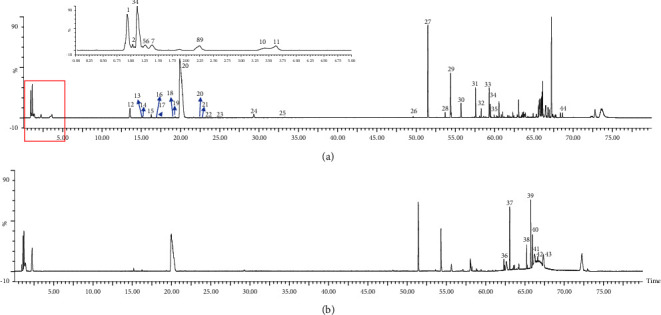
Chromatograms of Lycii Cortex in the positive ion mode (a) and negative ion mode (b).

The results of total ion chromatography (BPI) in positive and negative ion modes are shown in Figure 1. To determine the chemical composition of the compound, the exact mass of the compound was calculated according to the molecular formula, and full-scan chromatographic peaks such as [M − H]^−^, [M + H]^+^, and [M + Na]^+^ were extracted. The fragment ion information of the compound was obtained by MS/MS mass spectrometry and identified by reference to the target compound. Forty-four compounds were eventually identified (Table 3 and Figure 3). Among them, there were 23 alkaloids, 8 phenylpropanoids, 4 flavonoids, 2 organic acids, 1 quinone compound, and 6 unknown compounds. Eleven components were compared with standard substances and are listed in Table 3.

Ferulic acid was used as an example to illustrate the resolution process of organic acid compounds. In the negative ion mode, the parent ion of compound 12 was m/z 193.1130 [M − H]^−^, and its molecular formula was calculated to be C_10_H_10_O_4_. The parent ion removes a molecule of CH_3_ to produce a fragment ion at m/z 178.0274, a molecule of CO_2_ is removed to produce a fragment ion at m/z 134.0372, or the parent ion removes a molecule of CO_2_ and a molecule of CH_3_ to produce fragment ions at m/z 149.0616 and m/z 134.0372. Its retention time and mass spectrometry fragmentation behavior were consistent with those of the reference substance ferulic acid. The resolution process of alkaloid compounds was illustrated by the example of kukoamine B. In the negative ion mode, the precursor ion of compound 20 is m/z 529.2984 [M − H]^−^, and its molecular formula is calculated to be C_28_H_42_N_4_O_6_. The parent ion removed C_19_H_34_N_4_O_3_ to produce a fragment ion at m/z 165.0589, or the parent ion C_9_H_8_O_3_ generated a fragment ion at m/z 367.2708. Its retention time and mass spectrometry lysis behavior were consistent with those of the reference substance kukoamine B. According to the same method, other chemical constituents were identified by the polarity of the compound, the secondary fragment of its mass spectrum, the fragmentation rules, and the comparison with the literature data.

### 3.2. Identification of Prototype Components of LC in Rat Plasma, Urine, and Feces

By comparison with the 44 compounds identified by the research group in the previous stage, the prototype components in plasma, urine, and feces were screened. A total of 36 prototype components of LC were identified in rat plasma, urine, and feces, including 16 in plasma, 25 in urine, and 27 in feces. There are 10 common components in the prototype components of plasma, urine, and feces, one unique component in plasma, five unique components in urine, and nine unique components in feces. This reflects the metabolic law of the active ingredients in LC in vivo. The specific analytical results are listed in Table 3.

### 3.3. Component–Target–Disease Network Analysis

**Figure 4:**
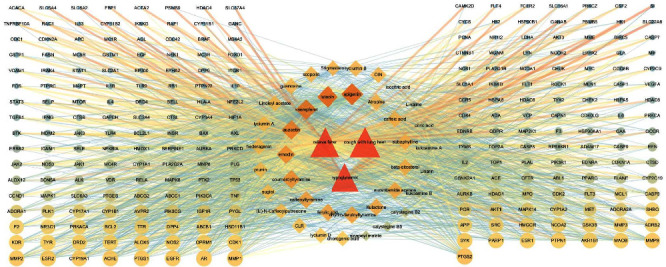
“Component–target–disease” network analysis of Lycii Cortex.

Based on the effectiveness of the Q-marker, we used network pharmacology technology to further analyze the relationships among drug components, targets, and diseases. Based on UPLC–Q-TOF-MS, 44 components in LC were identified. Combined with the components of LC in the TCMSP database, OB ≥ 30% and DL ≥ 0.18 were used as conditions to screen active components and targets. Through a literature search, 38 active components were initially identified. A total of 595 protein targets of 38 active components in LC were identified via the TCMSP, HERB, and UniProt databases. At the same time, a total of 854, 831, and 629 targets related to antipyretics, lung heat cough, and hypoglycemia, respectively, were identified. After deleting duplicate targets, 1695 disease targets related to LC efficacy were obtained. The 595 targets corresponding to LC were crossed with disease targets, and a total of 249 identical targets were obtained as potential targets for LC to exert its efficacy. Cytoscape 3.7.1 software was used to construct a component–target–disease network interaction map for topological analysis. A component–target–disease network diagram was constructed, as shown in Figure 4. The 38 active components in LC were based on 295 targets for the treatment of fever, lung heat cough, and hypoglycemia, including 290 nodes and 1679 edges, reflecting the interaction between the active components of LC and their efficacy. According to the degree value, 38 components, such as apigenin, may be the core active components of LC. The components entering the blood are usually the components through which the drug enters the body to exert its efficacy. Therefore, we compared the precursor components of LC in rat plasma. According to the specificity and measurability of the components, a total of 11 components, such as kukoamine A, kukoamine B, scopolin, kaempferol, guanosine, emodin, apigenin, ferulic acid, caffeic acid, chlorogenic acid, and luteolin, were screened as potential Q-markers of LC. This study lays a foundation for the subsequent selection of active ingredients as preparation components for content determination in LC.

### 3.4. Determination of the Content of Different Batches of LC

#### 3.4.1. Method Validation

The mass concentration of 11 components showed a good linear relationship with the peak area, *R*^2^ ≥ 0.99, as shown in Table 4. The relative retention time RSD of the 11 main components was 1.47%–1.98%, and the results showed that the precision of the instrument was good. The RSD of the contents of 11 main components was 0.39%–11.63%. The results showed that the method had good repeatability. The RSD of the peak area of the 11 main components was between 1.43% and 2.92% within 24 h, which met the requirements of the 2020 edition of Chinese Pharmacopoeia. The results showed that the method had good stability. The average recovery of the 11 main components was 95.43%–104.74%, and the RSD was 1.85%–2.76%, indicating that the recovery rate of the established content determination method was good.

#### 3.4.2. Content Determination

**Figure 5:**
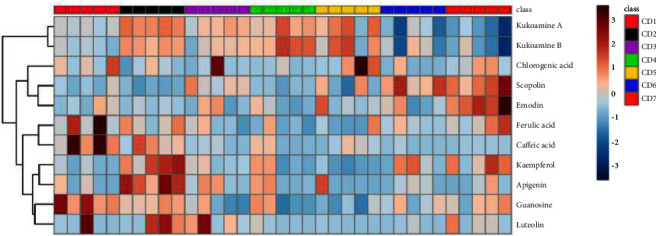
Heatmap analysis of the contents of 11 index components in Lycii Cortex.

The samples of 35 batches of LC from different origins were prepared and analyzed. The contents of kukoamine A, guanosine, kukoamine B, chlorogenic acid, scopolin, caffeic acid, ferulic acid, kaempferol, luteolin, apigenin and emodin in different batches of LC samples were calculated. The measurement results are shown in Table 5 and Figure 5. The contents of kukoamine A and kukoamine B were relatively high, with mass fractions ranging from 1.24 mg/g to 17.12 mg/g and from 5.25 mg/g to 67.35 mg/g, respectively. The mass fraction of scopoline ranged from 0.05 to 0.18 mg/g. The mass fraction of guanosine ranged from 0.02 to 0.83 mg/g. The mass fraction of ferulic acid ranged from 0.012 to 0.220 mg/g. The mass fraction of caffeic acid ranged from 0.012 to 0.460 mg/g. The mass fraction of chlorogenic acid ranged from 0.220 to 0.425 mg/g. However, the contents of kaempferol, emodin, apigenin, and luteolin were very low and could not be detected in some batches. Therefore, a variety of active ingredients can be controlled simultaneously in the quality control of medicinal materials to reflect the quality of medicinal materials more comprehensively.

### 3.5. High-Quality Production Area Analysis

**Figure 6:**
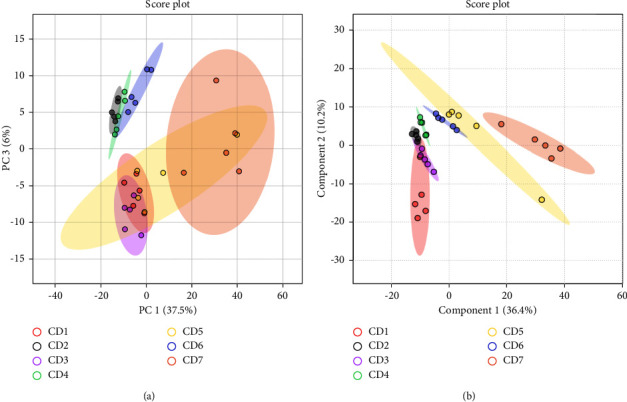
PCA and PLS-DA score plots of 35 batches of Lycii Cortex. (a) PCA diagram and (b) PLS-DA diagram.

**Figure 7:**
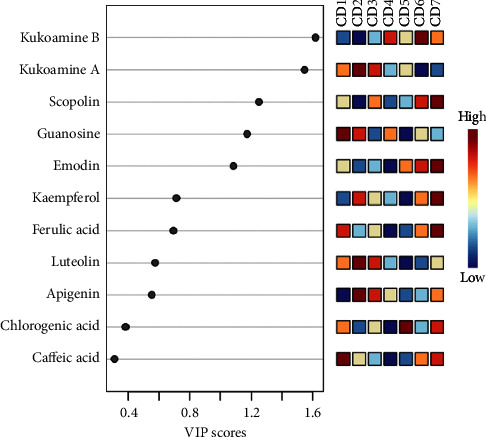
VIP score of 35 batches of Lycii Cortex.

To directly compare different origins to the differences in the chemical composition of the LC, PCA and PLS discriminant analysis (PLS-DA) were performed on LC in 35 different regions using the MetaboAnalyst website (https://www.metaboanalyst.ca/). According to the PCA score chart, the different producing areas can be divided into their own categories. Among them, in Chengcheng County, Weinan City, Shaanxi Province; Xinmi County, Zhengzhou City, Henan Province; and Wenxi County, Yuncheng City, Shanxi Province, the difference in ground quality in LC is small. It can be seen from the PLS-DA diagram that the analysis results are consistent with the PCA results, but the crossover of each origin is significantly narrowed, as shown in Figure 6. A VIP value > 1.0 indicated a significant effect. The differences in the contents of five components kukoamine A, kukoamine B, scopolin, guanosine, and emodin may be one of the reasons for the differences in the quality of the LC medicinal materials. The results are shown in Figure 7.

## 4. Discussion

Due to the complex situation of multiple sources and multiple production areas of Chinese medicinal materials, the quality of Chinese medicinal products varies greatly, especially the amount of active ingredients. Therefore, in 2016, the academician Changxiao Liu proposed a Q-marker for TCM, which is used for the quality control of TCM decoction pieces for harvesting, processing, and preparation. According to the 2020 edition of the Chinese Pharmacopoeia, the source of LC is the dried root bark of *Lycium chinense* Mill. or *Lycium barbarum* L., and there is no specific method for determining its content. Therefore, it is urgent to establish an efficient, sensitive, and reliable modern analysis method to realize the overall quality control of LC. The material chosen for our study was *Lycium chinense* Mill. To clarify the pharmacodynamic basis of LC, it is necessary to study the metabolic process of its main active ingredients in vivo. In recent years, many scholars have shown that blood components may be effective drug components. Compared with those in plasma, urine, and feces, there are more metabolites in plasma, urine, and feces. The comprehensive analysis of metabolites in serum, urine, and feces can help researchers better understand the metabolism and excretion process of active components in LC in vivo. In this study, 44 chemical components were identified in LC. UPLC–Q-TOF-MS analysis was subsequently performed on plasma, urine, and feces samples collected from rats after intragastric administration of LC. A total of 16 prototype components were identified in plasma, 25 prototype components in urine, and 27 prototype components in feces. Kaempferol, apigenin, ferulic acid, chlorogenic acid, and luteolin can be detected in plasma, urine, and feces. After absorption by the gastrointestinal tract, some of them enter the systemic circulation and are excreted in the urine, and some of them enter the small intestine through bile secretion and are excreted in the feces. Scopolin, emodin, and caffeic acid were detected in plasma and urine and were excreted mainly through the kidney after metabolism. Kukoamine A and kukoamine B were detected in plasma and feces. After metabolism, they are mainly absorbed into the blood through the small intestine or excreted via the feces. Guanosine is only detected in the blood and is mainly absorbed into the blood through the gastrointestinal tract after metabolism. The pharmacodynamic material basis of LC in vivo was preliminarily elucidated. Using the Q-marker method to further determine the quality of LC, we established a component–target–disease network through network pharmacology technology and exploited a method for determining the levels of 11 components, such as kukoamine A, via liquid chromatography‒mass spectrometry. The contents of 35 batches of LC from different producing areas were determined for the screening of high-quality LC-producing areas.

This study determined the levels of 11 indicator components, including kukoamine A, kukoamine B, scopolin, kaempferol, guanosine, emodin, apigenin, ferulic acid, caffeic acid, chlorogenic acid, and luteolin, in the LC from different producing areas. The 35 batches of LC from different producing areas were determined to meet the “Q-marker measurability.” The “effect component” (blood component) in the blood is the final link of the quality transfer system, and it is also an important basis for the determination of Q-markers of TCM. Eleven quantitative components in LC, including kukoamine A, kukoamine B, scopolin, kaempferol, guanosine, emodin, apigenin, ferulic acid, caffeic acid, chlorogenic acid, and luteolin, are blood components that meet the requirements of “Q-marker traceability.” The selected indicator components all have certain pharmacological effects on LC. A total of 38 active components in LC were screened out based on 295 targets for the treatment of fever, lung heat cough, and hypoglycemia by network pharmacology. Kukoamine A and kukoamine B are more abundant in the cuticle. The two are the isomers of spermine alkaloids and the main active components of LC, which have pharmacological effects, such as antioxidation [11], anti-inflammation [12], neuroprotection [13], lowering blood pressure [14], regulating blood lipids [15], and preventing Parkinson's disease [16]. Liu Wenwu [14] showed that in a rotenone-induced PC12 cell Parkinson's disease model, kukoamine A can reduce ROS and MDA levels and increase SOD activity to resist oxidative stress, thus playing an anti-Parkinson's role. In addition, kukoamine A improved gross motor function and neuronal activity in a Parkinson's disease model, increased the number of tyrosine hydroxylase (TH)-positive cells in the substantia nigra (SN) and striatum (Str), and decreased the expression of alpha-synuclein in the brain. The inhibition of apoptosis and enhancement of autophagy have protective effects on neurotoxin-induced PD [10]. Some scholars have shown that preconditioning with kukoamine A can alleviate the cell damage induced by *N*-methyl-D-aspartate (NMDA) and the dephosphorylation of Akt and GSK-3*β*, thus playing a neuroprotective role [13]. Kadilya established a rat model of otitis media and reported that kukoamine A could alleviate inflammation in rats by reducing the levels of inflammation-related cytokines, such as interleukin-4 (IL-4), interferon-gamma (IFN-*γ*), and tumor necrosis factor-*α* (TNF-*α*), in a dose-dependent manner [15]. Caffeic acid, ferulic acid, and chlorogenic acid are polyphenols in the LC. Studies have shown that polyphenols in the LC have antioxidant effects and can significantly increase the glutathione content and SOD activity in HSF cells damaged by H_2_O_2_ [16, 17]. An anti-inflammatory test of LPS-stimulated RAW264.7 cells by Xue Qiang showed that scopolin had a good anti-inflammatory effect. Pan et al. [18] reported that scopolin can reduce IL-6, VEGF, and FGF-2 expression in rat synovial tissues, which can reduce the clinical symptoms of AIA in rats by inhibiting inflammation and angiogenesis. Chlorogenic acid is metabolized into caffeic acid and ferulic acid in vivo and has various pharmacological effects, such as antioxidant, anti-inflammatory [19–22], antitumor [23], and neuroprotective effects. Flavonoids such as kaempferol, luteolin, apigenin, and emodin have pharmacological effects such as anti-inflammatory, antioxidant, and antidepressant effects, but their contents in the LC are relatively low. The above 11 components were confirmed to have “Q-marker effectiveness.”

Therefore, from the perspectives of “measurability,” “traceability,” and “effectiveness,” kukoamine A, kukoamine B, scopolin, kaempferol, guanosine, emodin, apigenin, FA, caffeic acid, chlorogenic acid, and luteolin were analyzed. Kukoamine A, kukoamine B, scopolin, guanosine, and emodin are the main differential components in LC and can be used to screen for high-quality medicinal materials and production areas of LC. Kukoamine A and kukoamine B met the requirements of the “five principles” and could be used as Q-markers for LC. It can be used for quality control, which provides a theoretical basis for the establishment of a comprehensive LC quality evaluation method and lays a foundation for subsequent related mechanism research.

## 5. Conclusion

In summary, this experiment established an LC-MS analysis method for the identification of LC chemical components and in vivo prototype components. Through network pharmacology research and content determination analysis, two potential marker system Q-markers of LC were determined based on the principles of traceability, specificity, effectiveness, and measurability: kukoamine A and kukoamine B, and the high-quality production area of LC was determined to be Chengcheng County, Weinan City, Shaanxi Province. This system can comprehensively and systematically reflect its quality and provide a reference for the further study of LC quality standards and the development of medicinal materials in the future.

## Figures and Tables

**Table 1:** Mass spectrum parameters of the 11 index components.

No.	RT (min)	Analytes	MRM				
			Parent	Daughters	Cone voltage	Collision energy	Ion mode
1	0.79	Kukoamine A	531.30	222.10	47	11	+
2	1.03	Guanosine	284.10	152.10	46	22	+
3	1.77	Kukoamine B	531.30	222.10	47	11	+
4	2.07	Chlorogenic acid	352.70	190.60	36	34	−
5	2.14	Scopolin	354.74	193.07	42	14	+
6	2.37	Caffeic acid	178.95	134.94	46	18	−
7	3.04	Ferulic acid	192.80	133.20	36	16	−
8	3.89	Kaempferol	286.95	153.02	42	32	+
9	3.89	Luteolin	284.90	133.00	50	20	−
10	4.34	Apigenin	268.80	116.60	50	20	−
11	6.52	Emodin	269.15	225.07	46	26	−

Abbreviations: MRM, multiple reaction monitoring; RT, retention time.

**Table 2:** Information on 35 batches of Lycii Cortex.

No.	Batch number	Producing area	Remark
1	DGP-20180806	Xinmi County, Zhengzhou City, Henan Province	CD1
2	DGP-20180807		
3	DGP-20180808		
4	DGP-20180809		
5	DGP-20180810		

6	DGP-181107-1	Ningxia Province	CD2
7	DGP-181107-2		
8	DGP-181107-3		
9	DGP-181107-4		
10	DGP-181107-5		

11	DGP-20180811	Wenxi County, Yuncheng City, Shanxi Province	CD3
12	DGP-20180812		
13	DGP-20180813		
14	DGP-20180814		
15	DGP-20180815		

16	DGP-20180801	Pucheng County, Weinan City, Shaanxi Province	CD4
17	DGP-20180802		
18	DGP-20180803		
19	DGP-20180804		
20	DGP-20180805		

21	DGP-20181027-201	Chengcheng County, Weinan City, Shaanxi Province	CD5
22	DGP-20181027-202		
23	DGP-20181027-203		
24	DGP-20181027-204		
25	DGP-20181027-205		

26	DGP-20181027-301	Pucheng County, Weinan City, Shaanxi Province	CD6
27	DGP-20181027-302		
28	DGP-20181027-303		
29	DGP-20181027-304		
30	DGP-20181027-305		

31	DGP-20181027-101	Lintong District, Xi'an, Shaanxi Province	CD7
32	DGP-20181027-102		
33	DGP-20181027-103		
34	DGP-20181027-104		
35	DGP-20181027-105		

*Note:* The same origin is not classified as a class of suppliers.

**Table 3:** Chemical composition analysis of Lycii Cortex.

No.	RT (min)	Positive ions	Negative ions	Formula	Compounds	Blood	Urine	Fecal
1	0.93	284.1388	282.9585	C_10_H_13_N_5_O_5_	Guanosine∗	+	−	−

2	1.03	176.0907	—	C_7_H_13_NO_4_	Calystegine B2/B5	−	−	+

3	1.08	—	317.0526	C_18_H_22_O_5_	4-Hydroxy-coumaroyl-2-nonenoic acid	+	+	+

4	1.12	118.0864	—	C_5_H_11_NO_2_	l-Valine	+	+	+

5	1.21	—	387.1135	C_25_H_26_O_13_	Isomer of hydroxycinnamic acid -ferulic acid glucoside	−	−	+

6	1.26	365.1052	341.1081	C_15_H_18_O_9_	Hydroxycinnamic acid glucoside	−	−	+

7	1.38	365.1052	341.1081	C_15_H_18_O_9_	Isomer of hydroxycinnamic acid glucoside	−	−	+

8	2.21	215.0177	191.0191	C_6_H_8_O_7_	Isocitric acid	−	+	−

9	2.26	407.0399	405.027	C_6_H_8_O_7_	Citric acid	−	+	−

10	3.42	235.1196	—	—	Unknown	−	−	−

11	3.63	235.1196	—	—	Unknown	−	−	−

12	13.57	195.1130	—	C_10_H_10_O_4_	Ferulic acid∗	+	+	+

13	15.11	202.0998	—	—	Unknown	−	−	−

14	15.19	457.132	479.1398	C_21_H_22_O_10_	Prunin	−	−	−

15	16.26	251.1405	249.1243	C_13_H_18_N_2_O_3_	(E)-*N*-Caffeoylputrescine	−	+	−

16	16.42	207.1518	—	C_13_H_18_N_2_O_3_	Isomer of (E)-*N*-caffeoylputrescine	−	+	−

17	16.95	144.0821	—	C_17_H_19_NO_4_	Dihydro-caffeoyltyramine	−	+	+

18	19.37	—	179.0366	C_9_H_8_O_4_	Caffeic acid∗	+	+	−

19	19.98	531.3184	529.3038	C_28_H_42_N_4_O_6_	Kukoamine A∗	+	−	+

20	21.68	531.3163	529.2984	C_28_H_42_N_4_O_6_	Kukoamine B∗	+	−	+

21	23.04	355.0991	—	C_16_H_18_O_9_	Chlorogenic acid∗	+	+	+

22	24.56	529.3063/265.1543	—	C_28_H_40_N_4_O_6_/C_14_H_2_0N_2_O_3_	N1-Caffeoyl-N10-dihydrocaffeoylspermine/*N*-feruloyl putrescine subaphylline	−	−	−
						−	−	−

23	25.63	281.1692	—	—	Unknown	−	−	−

24	29.32	474.2598	472.2442	C_25_H_35_N_3_O_6_	N1, N10-bis (Dihydrocaffeoyl) spermidine	−	−	−

25	33.26	193.052	—	C_16_H_12_O_6_	5,7-Dihydroxy-4′-methoxyflavone	−	+	+

26	47.27	545.1991	—	C_29_H_44_N_4_O_6_	*N*-Dihydroferuoyl-*N*′-dihydrocaffeoylspermine	−	+	+

27	51.51	874.3758	872.3596	C_42_H_51_N_9_O_12_	Lyciumin A	+	+	+

28	53.73	542.7795	1082.5367	C_51_H_59_N_10_O_17_	Dimethyl lycium B	−	+	−

29	54.35	897.3904	895.377	C_44_H_52_N_10_O_11_	Lyciumin B	−	−	+

30	55.65	964.4244	962.4056	C_47_H_53_N_11_O_12_	Lyciumin D	−	−	+

31	57.56	—	285.1377	C_15_H_10_O_6_	Luteolin∗	+	+	+

32	58.28	287.1204	—	C_15_H_10_O_6_	Kaempferol∗	+	+	+

33	59.28	274.2743	—	C_17_H_17_NO_4_	Caffeoyltyramine	+	+	+

34	59.46	331.3022	—	C_17_H_14_O_7_	3,4-Dimethyl-quercetin	+	+	+

35	60.53	316.2866	—	C_18_H_21_NO_4_	Dihydro-feruloyltyramine	−	−	+

36	62.78	—	269.1557	C_15_H_10_O_5_	Apigenin∗	+	+	+

37	63.05	—	295.2295	C_15_H_23_N_2_O_4_	Sinapoyl putrescine	−	+	+

38	65.22	—	269.1559	C_15_H_10_O_5_	Emodin∗	+	+	−

39	65.73	—	339.2311	C_15_H_16_O_9_	Sinapoyl malate	−	+	−

40	65.88	—	282.2785	C_17_H_17_NO_3_	Isomer of coumaroyltyramine	−	+	+

41	65.97	—	354.2705	C_17_H_21_NO_4_	Scopolin∗	+	+	−

42	66.03	—	337.234	C_20_H_23_NO_4_	Dimethyl-*N*-feruloyltyramine	−	−	+

43	66.12	—	337.2941	C_20_H_23_NO_4_	Isomer of dimethyl-*N*-feruloyl tyramine	−	−	+

44	67.22	284.2967	—	C_17_H_17_NO_3_	Coumaroyltyramine	−	+	+

^∗^Compared with the standard solution.

**Table 4:** Standard curve, correlation coefficient, and linear range test results of 11 chemical components.

Compounds	Representative	*R* ^2^	Linear range (ng/mL)
Kukoamine A	*y* = 0.87*x* − 321.52	0.9918	26.26∼2363.76
Kukoamine B	*y* = 1.397*x* − 1598.4	0.9920	38.61∼3475.08
Scopolin	*y* = 369.56*x* + 20055	0.9982	37.44∼3369.24
Kaempferol	*y* = 2761.9*x* + 324688	0.9955	28.64∼2577.87
Guanosine	*y* = 10.239*x* + 158930	0.9908	33.82∼3043.87
Emodin	*y* = 436.2*x* + 248471	0.9932	39.04∼3513.60
Apigenin	*y* = 19.194*x* + 2291	0.9959	35.12∼3160.80
Ferulic acid	*y* = 0.5171*x* − 214.94	0.9924	38.15∼3433.32
Caffeic acid	*y* = 347.02*x* − 147558	0.9927	38.62∼3475.53
Chlorogenic acid	*y* = 17.354*x* + 1781.3	0.9905	36.53∼3287.25
Luteolin	*y* = 17.974*x* + 997.94	0.9996	35.24∼3171.78

**Table 5:** Contents of 11 index components from Lycii Cortex.

Batch number	Kukoamine A (mg/g)	Kukoamine B (mg/g)	Scopoline (mg/g)	Kaempferol (μg/g)	Guanosine (mg/g)	Emodin (μg/g)	Apigenin (μg/g)	Ferulic acid (mg/g)	Caffeic acid (mg/g)	Chlorogenic acid (mg/g)	Luteolin (μg/g)
DGP-20181027-101	4.19	17.75	0.08	0.036	0.05	0.034	—	0.014	0.013	0.040	0.0134
DGP-20181027-102	7.82	33.68	0.10	0.021	0.08	0.061	0.01	0.014	0.016	0.079	0.0002
DGP-20181027-103	4.11	16.47	0.08	0.023	0.10	0.049	0.05	0.014	0.014	0.077	0.0048
DGP-20181027-104	3.10	13.08	0.08	0.040	0.06	0.039	0.11	0.015	0.014	0.071	0.0043
DGP-20181027-105	1.24	5.25	0.06	0.021	0.07	0.041	0.01	0.014	0.012	0.022	0.0004
DGP-20181027-201	10.74	37.99	0.13	0.027	0.18	0.074	0.35	0.015	0.014	0.073	0.0016
DGP-20181027-202	14.45	57.67	0.09	0.013	0.14	0.025	—	0.014	0.019	0.085	0.0004
DGP-20181027-203	15.56	60.89	0.06	0.007	0.37	0.038	—	0.013	0.030	0.176	—
DGP-20181027-204	11.07	42.86	0.18	0.017	0.10	0.030	0.01	0.014	0.027	0.425	—
DGP-20181027-205	12.45	43.64	0.08	0.014	0.32	0.031	—	0.020	0.019	0.182	—
DGP-20181027-301	6.92	30.77	0.11	0.014	0.18	0.032	0.01	0.015	0.014	0.047	—
DGP-20181027-302	2.69	12.27	0.10	0.035	0.11	0.032	0.05	0.015	0.015	0.070	0.0006
DGP-20181027-303	8.71	37.31	0.10	0.062	0.10	0.004	0.11	0.015	0.014	0.047	0.0056
DGP-20181027-304	7.42	31.41	0.10	0.028	0.18	0.010	0.08	0.014	0.014	0.041	—
DGP-20181027-305	3.54	16.25	0.10	0.013	0.11	0.018	—	0.013	0.013	0.032	—
DGP-20180801	11.31	45.26	0.10	0.061	0.69	0.049	0.24	0.014	0.029	0.074	0.0097
DGP-20180802	11.51	45.66	0.10	0.059	0.65	0.036	0.27	0.020	0.029	0.077	0.0023
DGP-20180803	17.12	67.35	0.09	0.012	0.07	0.017	0.01	0.014	0.014	0.130	—
DGP-20180804	14.18	62.75	0.11	0.024	0.19	0.012	0.01	0.015	0.014	0.080	0.0006
DGP-20180805	14.82	62.40	0.11	0.018	0.19	0.022	0.03	0.013	0.014	0.085	—
DGP-181107-1	12.37	43.89	0.08	0.059	0.42	0.021	0.45	0.020	0.018	0.030	0.0013
DGP-181107-2	12.57	46.62	0.07	0.039	0.49	0.033	0.36	0.017	0.046	0.083	0.0011
DGP-181107-3	13.99	49.12	0.09	0.090	0.61	0.026	0.24	0.015	0.039	0.144	0.0407
DGP-181107-4	14.40	50.71	0.08	0.098	0.76	0.032	0.61	0.018	0.027	0.095	0.0495
DGP-181107-5	11.28	41.00	0.07	0.087	0.50	0.022	0.39	0.020	0.026	0.059	0.0205
DGP-20180806	8.01	30.82	0.08	0.027	0.83	0.017	0.15	0.015	0.020	0.097	0.0017
DGP-20180807	5.60	20.76	0.05	0.010	0.19	0.016	—	0.019	0.045	0.041	0.0005
DGP-20180808	7.55	28.20	0.07	0.025	0.70	0.013	0.10	0.015	0.028	0.086	0.0407
DGP-20180809	5.32	20.66	0.05	0.011	0.18	0.030	—	0.022	0.045	0.042	—
DGP-20180810	6.39	21.38	0.07	0.013	0.29	0.025	—	0.014	0.027	0.131	—
DGP-20180811	8.18	27.82	0.13	0.033	0.25	0.015	0.07	0.016	0.016	0.047	0.0159
DGP-20180812	8.26	30.07	0.08	0.034	0.33	0.039	0.20	0.017	0.017	0.062	0.0366
DGP-20180813	10.61	40.22	0.10	0.023	0.16	0.056	0.23	0.014	0.020	0.281	0.0016
DGP-20180814	11.29	39.83	0.12	0.026	0.04	0.009	0.05	0.015	0.014	0.068	0.0145
DGP-20180815	10.98	40.87	0.11	0.028	0.02	0.021	0.04	0.012	0.014	0.071	0.0034

## Data Availability

The data that support the findings of this study are available from the corresponding author upon reasonable request.

## Nomenclature

LC: Lycii Cortex
Q-marker: Quality markers
UPLC–Q-TOF-MS/MS: Ultrahigh-performance liquid chromatography with quadrupole time-of-flight mass spectrometry
UPLC–MS/MS: A rapid and sensitive analytical method for ultrahigh-performance liquid chromatography coupled with mass spectrometry
PCA: Principal component analysis
TCM: Trational Chinese medicine
Goqi: *Lycium chinense Mill.*
Ningxia Goqi: *Lycium barbarum* L.
HPLC: High-performance liquid chromatography
ESI: Electrospray ionization
TCMSP: Traditional Chinese Medicine Systems Pharmacology Database and Analysis Platform
OB: Oral bioavailability
DL: Drug likeness
BPI: Base peak ion
RSD: Relative standard deviation
QC: Quality control
RE: Relative error
RSD: Relative standard deviation
PLS-DA: Partial least squares discriminant analysis
ROS: Reactive oxygen species
MDA: Malondialdehyde
SOD: Superoxide dismutase
TH: Tyrosine hydroxylase
SN: Substantia nigra
Str: Striatum
NMDA: *N*-Methyl-D-aspartate
IL-4: Interleukin-4
IFN-*γ*: Interferon-gamma
TNF-*α*: Tumor necrosis factor-*α*
VEGF: Vascular endothelial growth factor
FGF-2: Fiber growth factor 2
FDA: Food and Drug Administration
LLOQ: Lower limit of quantification
